# Recent Advances in Electrochemical Biosensors for Neurodegenerative Disease Biomarkers

**DOI:** 10.3390/bios15030151

**Published:** 2025-02-28

**Authors:** Mingyu Bae, Nayoung Kim, Euni Cho, Taek Lee, Jin-Ho Lee

**Affiliations:** 1 Department of Information Convergence Engineering, Pusan National University, Yangsan 50612, Republic of Korea; mgbae@pusan.ac.kr (M.B.); skdud0822@pusan.ac.kr (N.K.); whdmsdl08@pusan.ac.kr (E.C.); 2 Department of Chemical Engineering, Kwangwoon University, Seoul 01897, Republic of Korea; 3 School of Biomedical Convergence Engineering, Pusan National University, Yangsan 50612, Republic of Korea; 4 Research Institute of Convergence of Biomedical Science and Technology, Pusan National University Yangsan Hospital, Yangsan 50612, Republic of Korea

**Keywords:** neurodegenerative disease, electrochemical biosensor, α-synucleins, amyloid-β, tau proteins

## Abstract

Neurodegenerative diseases, such as Parkinson’s disease (PD) and Alzheimer’s disease (AD), represent a growing global health challenge with overlapping biomarkers. Key biomarkers, including α-synucleins, amyloid-β, and Tau proteins, are critical for accurate detection but are often assessed using conventional methods like enzyme-linked immunosorbent assay (ELISA) and polymerase chain reaction (PCR), which are invasive, costly, and time-intensive. Electrochemical biosensors have emerged as promising tools for biomarker detection due to their high sensitivity, rapid response, and potential for miniaturization. The integration of nanomaterials has further enhanced their performance, improving sensitivity, specificity, and practical application. To this end, this review provides a comprehensive overview of recent advances in electrochemical biosensors for detecting neurodegenerative disease biomarkers, highlighting their strengths, limitations, and future opportunities. By addressing the challenges of early diagnosis, this work aims to stimulate interdisciplinary innovation and improve clinical outcomes for neurodegenerative disease patients.

## 1. Introduction

Neurodegenerative diseases are a leading cause of increasing morbidity and disability, particularly in aging populations, and they pose a significant global socioeconomic problem [1,2]. For example, Parkinson’s disease (PD) is one of the most common and fastest-growing neurodegenerative disorders, affecting 1–3% of the worldwide population over 60 [3,4]. The primary pathological feature of PD is the loss of over 80% of dopamine-producing neurons in the substantia nigra (SN), resulting in progressive impairments in motor functions, mental health, sleep, and pain regulation [5]. As symptoms worsen over time, the importance of early diagnosis and effective patient classification has become increasingly apparent to mitigate disease progression and enhance patient outcomes [6].

While α-synuclein is a key biomarker for PD, the molecular pathology of neurodegenerative diseases often overlaps significantly, complicating diagnosis [7,8]. For instance, amyloid-beta (Aβ) and Tau proteins, traditionally associated with Alzheimer’s disease (AD), are also found in cerebrospinal fluid (CSF) and are critical in diagnosing and monitoring AD [9,10]. In AD, amyloid-beta plaques and Tau neurofibrillary tangles are the hallmark pathologies [11,12]. However, these biomarkers can sometimes be detected in patients with mixed neurodegenerative conditions, complicating diagnosis and highlighting the importance of comprehensive biomarker profiling [13]. The shared presence of such biomarkers across diseases like PD and AD suggests that a multi-biomarker approach could be instrumental in early diagnosis and differentiation between these neurodegenerative conditions. This overlap highlights the need for a comprehensive biomarker profiling approach to enable early differentiation between PD, AD, and other neurodegenerative conditions. Such a multi-biomarker strategy could prove instrumental in improving diagnostic accuracy and tailoring personalized treatment plans for affected individuals. Although the traditional methods such as enzyme-linked immunosorbent assay (ELISA), polymerase chain reaction (PCR), and real-time PCR are mainly utilized for the detection of neurodegenerative disease biomarkers, they are invasive, slow, costly, and require highly specialized tools [14,15,16]. Thus, addressing the challenges of early detection and precise characterization, there is a critical need for accurate diagnostic methods supported by reliable biomarkers.

Recently, electrochemical biosensors have emerged as powerful tools for detecting biomarkers associated with neurodegenerative diseases [17]. The electrochemical biosensor, one of the types of biosensors, combines an analyte acceptance mechanism and an electrochemical transducer together, where the interaction between the target analyte and the transducer produces an electrochemical signal in the form of current, potential, resistance, or impedance. Electrochemical analysis has attracted a lot of attention from a diagnostic point of view due to its inherent high sensitivity, ease of miniaturization, integration into portable devices, and ability to operate in turbid solutions [18,19]. However, in the case of protein biomarkers, they can hinder charge transfer within the biosensor, potentially reducing its sensitivity [20]. Therefore, it is important to recognize that different electrochemical biosensing modalities come with their own sets of advantages and disadvantages. For example, amperometry sensors are highly sensitive and can detect ultra-low concentrations of biomarkers; however, they are prone to interference from other electroactive species in complex biological matrices [21,22,23,24]. Potentiometric sensors, on the other hand, offer simple designs and low power consumption but typically lack the sensitivity required for detecting low-abundance biomarkers [25,26]. Voltammetry sensors provide detailed electrochemical profiles and can facilitate multi-analyte detection, yet they often demand more complex instrumentation and careful optimization to minimize noise and interference [27,28]. Impedimetric sensors enable label-free and real-time monitoring, though their performance can be affected by variations in environmental conditions and sensor interface stability [29,30].

The integration of nanomaterials into these platforms has significantly improved their sensitivity, specificity, and practicality, enabling early and accurate detection of neurodegenerative disease biomarkers [31]. Nanomaterials offer distinct characteristics such as exceptional electrochemical properties, a large surface area, a faster charge transfer rate, and adjustable electrical conductivity, making them highly appealing for sensing applications, particularly in electrochemical sensing [32,33]. Moreover, their compatibility with other techniques creates potential for electrochemical sensors across a wide range of biomedical applications. In addition to the integration of nanomaterials, the size and shape of the sensor platforms play a critical role in determining the performance of electrochemical biosensors [34]. The size of the sensor platform influences its surface area, which directly affects the sensitivity and efficiency of biomarker detection [35]. Smaller platforms with a larger surface area allow for a higher density of functionalized sites, enabling more efficient interaction between the target biomarker and the sensor [36]. Moreover, the nanoscale size can enhance the sensor’s ability to detect low concentrations of biomarkers, making them ideal for early-stage diagnostics in neurodegenerative diseases [37]. The shape of the sensor platform is another key factor that impacts performance. Different shapes, such as spherical, rod-like, and disk-like, can affect the distribution of electrochemical signals and the interaction with target molecules [38,39]. For instance, nanorods or nanowires can provide a higher aspect ratio, promoting faster electron transfer and increasing the sensitivity of the sensor [40]. On the other hand, spherical nanoparticles might offer a more uniform distribution of functional groups but may have limitations in terms of surface area compared with elongated structures. The choice of shape should, therefore, be tailored to the specific application, balancing factors such as signal strength, stability, and ease of fabrication [41]. Together, the size and shape of electrochemical biosensor platforms significantly influence their performance, with nanomaterial integration further enhancing their capabilities [42]. Understanding and optimizing these physical characteristics will continue to be a crucial area for the development of next-generation biosensors, particularly in the field of neurodegenerative disease diagnostics.

**Figure 1:**
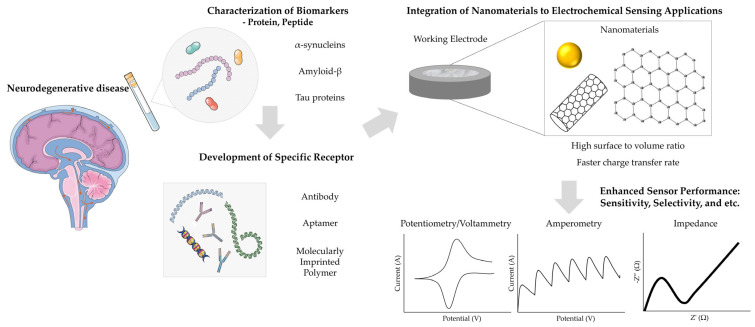
Operating principle of an electrochemical biosensor that detects disease through biological fluids. The figure was created using elements from Servier Medical Art, licensed under CC BY 4.0.

While numerous reviews have explored diagnostic platforms for various diseases, the growing prevalence of neurodegenerative conditions highlights the urgent need for focused investigation into electrochemical biosensors for the early and precise detection of their biomarkers [43]. In this review, we aim to present a comprehensive overview of recent advancements in electrochemical biosensors for neurodegenerative diseases, with an emphasis on key biomarkers such as α-synucleins, amyloid-β, and Tau proteins (Figure 1). This analysis will emphasize the transformative role of nanomaterials in enhancing biosensor performance, particularly in terms of sensitivity, specificity, and ease of application. Furthermore, we will highlight the strengths and limitations of electrochemical biosensors, along with their future challenges and opportunities for application in early diagnosis and monitoring. Through this focused discussion, we aim to foster interdisciplinary collaboration and drive innovation, ultimately advancing diagnostic capabilities and improving outcomes for individuals affected by neurodegenerative diseases.

## 2. Electrochemical Biosensor for α-Synucleins

Alpha-synuclein (α-synuclein) is widely recognized as a critical biomarker for neurodegenerative disorders, including PD and multiple system atrophy (MSA), and is also implicated in the pathophysiology of AD [44]. In PD, the progressive degeneration of dopaminergic neurons in the substantia nigra pars compacta, coupled with the pathological accumulation of α-synuclein in Lewy bodies [45,46]. This protein plays a pivotal role in regulating dopamine synthesis by interacting with tyrosine hydroxylase (TH), inhibiting its activity, and reducing dopamine levels, leading to the progressive loss of motor function [47]. In MSA, α-synuclein accumulates predominantly in oligodendrocytes, forming glial cytoplasmic inclusions (GCIs) that differentiate its pathology from PD [48,49]. Furthermore, in AD, although amyloid-beta and Tau proteins are the primary pathological markers, α-synuclein often co-aggregates with these proteins, potentially exacerbating neurodegenerative processes and contributing to synaptic dysfunction and cognitive decline [50,51]. The critical role of α-synuclein across these neurodegenerative disorders underscores the need for advanced detection methods to facilitate early diagnosis and monitor disease progression. Below, the advancements in this field are explored, highlighting innovative approaches researchers have employed to enhance biosensor performance.

Recent advances in biosensor technologies, particularly electrochemical platforms incorporating nanomaterials, have significantly enhanced the sensitivity and specificity of α-synuclein detection. This improvement is attributed to the unique properties of nanomaterials, including their high surface area, conductivity, and biocompatibility. Among these approaches, the integration of gold nanoparticles (AuNPs) into biosensor designs has significantly improved the sensitivity and specificity of α-synuclein detection. For instance, Karaboga et al. developed a disposable neuro-biosensor system using AuNPs and polyglutamic acid (PGA)-modified indium tin oxide (ITO) electrodes for the detection of α-synuclein in CSF [52]. This may aid in the diagnosis and prognosis of the disease by precisely measuring the amount of α-synuclein in the cerebrospinal fluid (CSF). ITO substrate, known for its high conductivity, polarizable surface, and stability under physiological conditions, was utilized as a reliable and efficient electrode. The electrode surface was further optimized with electropolymerized glutamic acid, facilitating robust covalent bonding with anti-α-synuclein antibodies. The PGA modification activated carboxylic acid terminal groups on AuNPs and improved molecular recognition and binding efficiency. When the peak potential difference (ΔE_p_) was compared by calculating the redox peak potential difference (ΔE_p_ = E_pc_ − E_pa_), the peak current separation of the ITO electrode modified with polyglutamic acid polymer and AuNPs decreased to 0.31 V. This indicates that AuNPs and PGA provide conductive bridges to accelerate electron transfer. The analytical performance of this biosensor was demonstrated to have a linear detection range of 4–2000 pg/mL by comparing various concentrations and electron transfer resistance using electrochemical impedance spectroscopy (EIS), and the limit of detection (LOD) and limit of quantitation (LOQ) was confirmed to be 0.135 pg/mL and 0.45 pg/mL, respectively. The system maintained full selectivity for the target antigen for 6 weeks, ignoring non-specific interactions. Additionally, it exhibited excellent reproducibility, storage stability, and minimal non-specific binding. The disposable design of the biosensor further underscores its potential for rapid clinical assessments, disease progression monitoring, and even patient self-assessment applications.

Similarly, Aminabad et al. present the development of a novel, highly sensitive, and specific electrochemical immunosensor for the quantification of α-synuclein protein in human biofluids [53]. The immunosensor was designed using a green-synthesized gold nanoparticle-supported dimethylglyoxime (AuNPs@DMGO) layer, prepared via a one-step electro-generation method, and electrodeposited onto a glassy carbon electrode (GCE) to provide a conductive and high-surface-area substrate. A sandwich immunosensor platform was constructed by immobilizing a biotinylated primary antibody (Ab1) specific to α-synuclein on the AuNPs-modified GCE surface, followed by conjugation with a secondary antibody (HRP-Ab2) to form the immuno-complex. The engineered AuNPs layer demonstrated excellent electrochemical properties, including enhanced conductivity and abundant active functional groups for antibody immobilization. As a result, this design enabled precise and sensitive detection of α-synuclein with a linear range of 4–64 ng/mL and a limit of detection of 4 ng/mL in both standard buffers and human plasma samples. When tested every 2 h for repeatability of this sensor, peak currents for both oxidation and reduction peaks decreased by 42% until 4 h. Building on these advancements, the same group developed an electrochemical immunosensor by incorporating gold nanoparticle-modified graphene (AuNP-Gr) [14]. This hybrid material exhibited enhanced conductivity, increased surface area, and strong adsorption capabilities. The combination of AuNPs and graphene provides synergistic benefits that further improve the sensor’s sensitivity and selectivity. As a result, this sensor enables the detection of α-synuclein within a wider detection range of 4–128 ng/mL and a lower limit of detection of 4 ng/mL, offering significant improvements over the earlier design.

Further advancing ultrasensitive detection capabilities, a major innovation in electrochemical biosensor development is the use of nanocomposites, which combine multiple materials to leverage their individual properties [54,55]. Nanohybrids and hierarchical systems have gained significant attention in this area due to their enhanced properties compared with traditional monometallic or single-element sensors [56,57]. Unlike single-element sensors, which often exhibit limitations in terms of stability, sensitivity, and selectivity, nanohybrids incorporate various materials such as metals, semiconductors, and carbon-based nanomaterials, while each contributes its unique advantages [58]. These hybrid systems offer superior electrochemical properties, including higher surface area, improved charge transfer rates, and enhanced catalytic activity, making them more effective for detecting low concentrations of biomarkers [59,60,61]. For example, hierarchical structures with multiple levels of porosity and unique geometric configurations enable more efficient interactions with analytes, thus enhancing detection performance even in complex biological samples [62]. These combined characteristics make nanohybrids and hierarchical systems particularly promising for the design of next-generation electrochemical biosensors, offering a distinct advantage over monometallic sensors in terms of versatility, performance, and applicability across diverse sensing environments. A notable example of this approach is demonstrated by Mari et al. [63]. The sensor system incorporated zinc oxide nanostars (ZnO NSs) decorated with AuNPs and functionalized with anti-α-synuclein antibodies through electro-polymerized glutamic acid. ZnO NWs provided excellent biocompatibility and conductivity, while gold nanostars (AuNSs) amplified the signal through their extensive surface area. The charge transfer resistance exhibited a linear relationship with α-synuclein concentrations in the range of 0.5–10 pg·mL^−1^, with a remarkably low LOD of 0.08 pg·mL^−1^. The sensor has been developed into a tool that could accelerate noninvasive early identification. The sensor demonstrated excellent reproducibility (5% variation), stability (90% retention after two months), and minimal matrix effects when tested with plasma samples. However, the complex synthesis and high cost of ZnO surface modification require further optimization.

**Figure 2:**
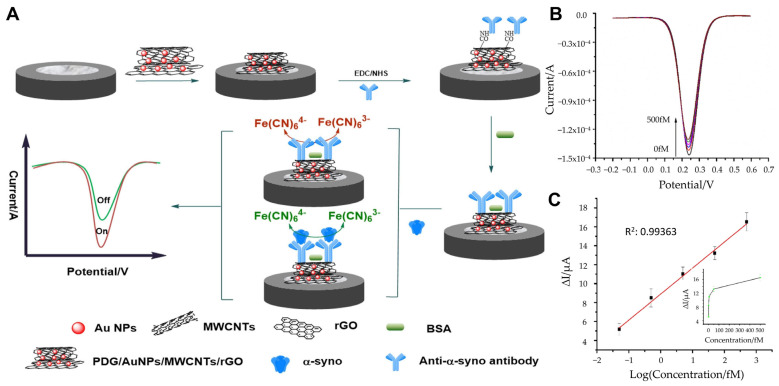
(**A**) Schematic representation of a α-synuclein detection with electrochemical immunosensor. (**B**) SWV responses for different concentrations of α-synuclein: 0.00, 0.05, 0.50, 5.00, 50.00, 500.00 fM. (**C**) Linear plot of the immunosensor for different concentrations of α-synuclein. (**A**–**C**): reproduced with permission from [64], published by Microchemical Journal 2020.

Furthermore, the integration of metal nanoparticles with carbon-based nanostructures has been shown to significantly improve the stability and sensitivity of electrochemical sensors. In this regard, Tao et al. utilized a nanocomposite composed of poly(D-glucosamine) (PDG), AuNPs, multi-walled carbon nanotubes (MWCNTs), and reduced graphene oxide (rGO) for signal amplification (Figure 2A) [64]. The PDG/AuNP/MWCNT/rGO nanocomposite was immobilized on a glassy carbon electrode, where the carboxyl groups of MWCNTs enabled the covalent attachment of anti-α-synuclein antibodies. PDG facilitated the reduction in HAuCl_4_, preventing AuNP aggregation and enhancing the dispersion of AuNPs and rGO, thereby improving the electrical and film-forming properties of the nanocomposite. Using square wave voltammetry (SWV), the immunosensor demonstrated a broad linear detection range of 0.05–500 fM and a remarkably low limit of LOD of 0.03 fM (Figure 2B,C). The sensor exhibited excellent sensitivity, selectivity, and stability, effectively detecting ultra-trace concentrations of α-synuclein in human plasma samples. In triplicate analyses of plasma samples, the relative standard deviations (RSDs) of 1, 10, and 100 fM α-synuclein solutions were all determined to be less than 5% (1.19%, 1.48%, and 3.71%, respectively).

**Figure 3:**
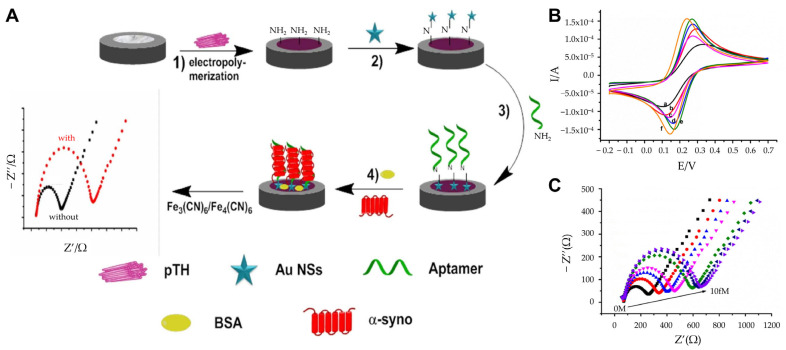
(**A**) Schematic representation of the aptasensor fabrication: (1) Electropolymerization of pTH onto the electrode; (2) deposition of AuNSs; (3) adsorption of aptamers; (4) blocking of non-specific binding with BSA. (**B**) CV characterization of the nanomaterial: (a) Bare GCE, (b) AuNPs-GCE, (c) AuNSs-GCE, (d) TH-GCE, (e) pTH-GCE, (f) pTH/AuNSs-GCE. (**C**) EIS response to varying α-synuclein concentrations with varying concentration (0 M to 10.00 fM), demonstrating a dynamic range and ultra-sensitive detection. (**A**–**C**): reproduced with permission from [65], published by Journal of Applied Electrochemistry 2021.

In parallel to immunosensors, aptasensors have emerged as promising alternatives for α-synuclein detection. Tao et al. developed a highly sensitive, label-free impedimetric aptasensor for detecting α-synuclein [65]. It was developed to analyze trace amounts of α-syno using non-invasive measurement methods to diagnose and treat diseases early and reduce the harm of human sampling. The platform incorporated polythionine (pTH) and AuNSs onto a GCE, utilizing their excellent electrical conductivity, rapid electron transfer capability, and synergistic signal amplification to enhance detection performance (Figure 3A). Cyclic voltammetry (CV) and EIS confirmed the stepwise assembly and enhanced conductivity of the pTH/AuNSs-modified GCE compared with bare or partially modified electrodes (Figure 3B). Measurements were conducted in 0.1 M PBS with 5 mM K_3_Fe(CN)_6_/K_4_Fe(CN)_6_. CV parameters: potential range of −0.2 V to +0.7 V, scan rate 100 mV/s. The sensor employed single-stranded DNA aptamers to recognize and bind α-synuclein specifically. This approach enabled ultra-sensitive detection with a remarkably low LOD of 0.07 aM and a dynamic range from 0.10 aM to 10 fM (Figure 3C). The aptasensor demonstrated reliable selectivity and good sensitivity, even in diluted human plasma samples (1:4000 dilution), with minimal matrix effects. To verify the reliability of the constructed aptasensor, plasma samples from five normal volunteers were tested. Each concentration was measured three times, and the average and RSD were calculated. The values of RSD were 4.32–4.56%. This demonstrates the operability of the aptasensor in real physiological media. The aptasensor’s simplicity, non-invasive measurement capability, and ultra-low LOD make it a promising tool for the early diagnosis of PD. Additionally, its adaptable design suggests potential applications in detecting other PD biomarkers, offering a robust platform for future diagnostic advancements.

In another aspect, Yao et al. extended detection capabilities to the photoelectrochemical (PEC) domain by integrating AuNPs, graphdiyne (GDY), and tungsten selenide (WSe_2_) nanoflowers using a strategy that combines aptamers and PEC assays to report signals with ultrahigh sensitivity and a wide response range [66]. GDY provided high conductivity, a large surface area, and reduced background signals, while WSe_2_ improved optoelectronic properties by minimizing electron–hole recombination and enhancing charge separation. The biosensor employed a dual-signal amplification strategy: (1) Cycle I converted α-synuclein into a high output of false-target DNA (FT), (2) Cycle II used FT to catalyze hairpin assembly on the AuNPs/GDY-modified electrode, capturing PEC nanoprobes composed of dopamine (DA), 4-mercaptophenyl boronic acid (MBA), and WSe_2_ (DA/MBA/WSe_2_). This innovative approach produced a high PEC signal with ultralow background noise (27.6 nA) and achieved an LOD of 3.3 aM with a dynamic range of up to 100 aM, demonstrating excellent selectivity and reproducibility (RSD = 3.7%). Additionally, the applicability and feasibility were verified through the sample standard addition method. The experimental results clearly showed that the RSD was between 2.36% and 4.02%, demonstrating that this biosensor has the potential for auxiliary clinical diagnosis. The sensor’s performance was validated in clinical-like settings, showing promise for early PD diagnosis and monitoring. Overall, the synergy between GDY and WSe_2_ nanoflowers, combined with the dual-signal amplification, established a robust platform for sensitive and selective α-synuclein detection, highlighting its potential for clinical applications in PD diagnostics.

Building on the diverse approaches for α-synuclein detection, targeting specific variants of the protein has also been explored to enhance diagnostic precision. For example, nitrated α-synuclein, a modification associated with oxidative stress and commonly observed in patients with Lewy body disorders, represents one such variant with significant clinical relevance. Zhang et al. developed an ultrasensitive electrochemical immunosensor that used gold nanocages (GNCs) as a sensing platform, which provided high surface area, conductivity, and biocompatibility [67]. This presented a novel approach in which a nitrated α-syn immunosensor utilizing GNCs was developed as a sensing platform, and anti-nitro-α-syn and anti-α-syn-modified MNPs were immobilized as signal amplifiers. Magnetic nanoparticles (MNPs) functionalized with anti-nitrated α-syn antibodies served as signal amplifiers, improving the sensitivity and selectivity of the assay. The sensor operates via a sandwich assay where GNCs capture anti-nitrated α-syn, while MNPs–Ab enhances the electrochemical signal. The combination of GNCs and MNPs led to a significant decrease in charge-transfer resistance, enabling more efficient electron transfer and improved signal output. This setup produced a linear response for nitrated α-syn concentrations ranging from 1 to 1000 ng/mL with an LOD of 310 pg/mL. The immunosensor demonstrated excellent stability, high selectivity, and the ability to detect nitrated α-syn in blood samples. Additionally, the applicability of the biosensor was tested by measuring the levels of nitrated α-syn in diluted serum samples from healthy donors (*n* = 8) and PD patients (*n* = 8) and comparing the Δ R ct values with those of clinical samples. We found that the Δ R ct responses due to serum nitrated α-syn concentrations in PD patients were significantly different, suggesting that this electrochemical immunoassay has potential clinical application value. This approach highlights the potential of specialized sensors to not only advance PD diagnostics but also support personalized treatment strategies by distinguishing between different α-synuclein variants. The recent research on electrochemical biosensors for α-synucleins is compared in Table 1.

## 3. Electrochemical Biosensor for Amyloid-β

Amyloid-β (Aβ) peptide is generated through the proteolytic cleavage of amyloid precursor protein (APP), a transmembrane protein. Pathological accumulation of Aβ occurs primarily in the brain, cerebrovascular system, and skeletal muscle [70]. In PD patients, lower CSF Aβ levels correlate with increased cortical amyloid-β deposition, which is associated with heightened cognitive decline [71,72]. In AD, Aβ forms extracellular neurotoxic fibrils that can diffuse back into the CSF, making it a key biomarker for detecting pathological features. Aβ accumulation induces synaptic degeneration, disrupts intracellular calcium (Ca^2+^) homeostasis, exacerbates excitotoxicity, and contributes to the formation of amyloid plaques and neurofibrillary tangles (NFTs), which are hallmarks of AD progression [73,74,75]. To this end, various biosensing platforms have been developed for sensitive and specific detection of Aβ species, leveraging advanced materials and electrochemical techniques.

A major innovation in electrochemical biosensor development is the use of nanocomposites, which combine multiple materials to leverage their individual properties. Qin et al. introduced a hierarchical design integrating gold dendrites (AuD), poly(pyrrole-3-carboxylic acid) [PPy-3-COOH], and cellular prion protein (PrPC) for ultra-sensitive detection of Aβ oligomers (AβOs) [76]. The AuD substrate, synthesized electrochemically through chronoamperometry, provided extensive active sites and high conductivity due to its dendritic structure with micrometer-scale trunks and nanometer-scale branches. PPy-3-COOH, a conductive polymer, was polymerized onto the AuD surface, offering a platform for covalent immobilization of PrPC through amide bond formation. PrPC, known for its high binding affinity for AβOs, enhanced the sensor’s specificity and selectivity. The biosensor achieved an exceptionally low detection limit of 1 aM and demonstrated a linear impedance response for AβO concentrations ranging from 10^−9^ to 10^−2^ nM. The relative standard deviation (RSD) for independent measurements in mouse brain samples at various concentrations was less than 4%. This hierarchical approach illustrates how structural and functional optimization can be synergistically employed to set new benchmarks in biosensor sensitivity.

**Figure 4:**
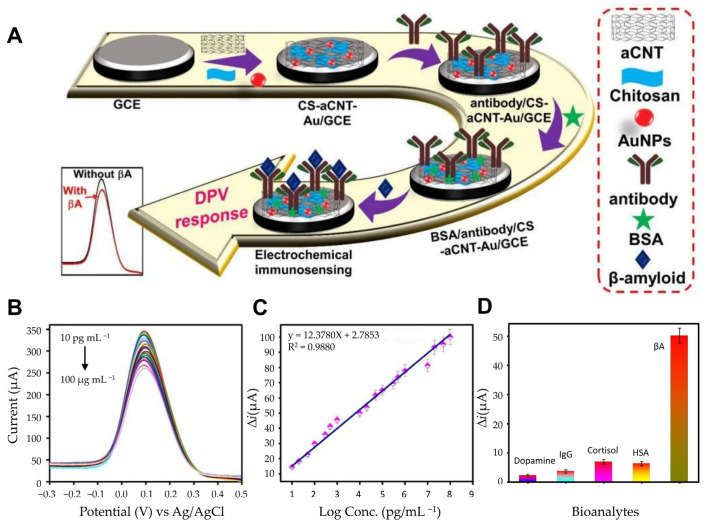
(**A**) Schematic of the fabrication of the BSA/antibody/CS-aCNT-Au immunosensor. (**B**) DPV curves of BSA/antibody/CS-aCNT-Au/GCE for Aβ in PBS. (**C**) The related calibration curve of the immunosensor. (**D**) Selective specificity of the immunosensor for Aβ for various biological analytes. (**A**–**D**): reproduced with permission from [77], published by Biosensors 2022.

Carbon-based materials also play a pivotal role in biosensor innovation due to their exceptional electrical conductivity, biocompatibility, and ease of surface modification. Ranjan et al. developed an electrochemical immunosensor platform utilizing a chitosan (CS) and carbon nanotube (CNT) composite functionalized with AuNPs, forming a CS-aCNT-Au nanocomposite (Figure 4A) [77]. This composite capitalizes on CNTs’ large surface area and graphene-like properties, with the added conductivity enhancement from AuNPs. The active surface area of CS-aCNT/GCE was measured to be 8.594 × 10^−2^ cm^2^, which increased to 9.735 × 10^−2^ cm^2^ with the addition of AuNPs. Chitosan provided functional amine and hydroxyl groups for antibody immobilization and ensured uniform coating on the electrode surface. Quantitative analysis of Aβ peptides via DPV demonstrated high sensitivity, with a linear detection range from 10.0 pg/mL to 100.0 pg/mL and a detection limit of 0.87 pg/mL (Figure 4B,C). The developed sensor was analyzed with various biological analytes, and the results showed that the current change for Aβ was significantly higher compared with other analytes. This indicates minimal interference and confirms that the sensor can selectively detect Aβ (Figure 4D). To investigate the selectivity, we tested it against various bioanalytes. The current changes for other bioanalytes were much smaller, so interference could be ignored, demonstrating the high selectivity of the immunosensor. This system exemplifies how carbon-based materials and nanocomposites synergize to produce highly efficient biosensors. Additionally, graphene derivatives, particularly reduced rGO, have also been extensively used in biosensing due to their high electrical conductivity, biocompatibility, and large surface-to-volume ratio. Sethi et al. developed a dual-layer graphene/rGO screen-printed electrode (SPE) for the detection of Aβ1-42 [78]. This allows rapid and label-free detection of Aβ. By combining graphene and rGO, this biosensor maximized reactive sites and conductivity, which is essential for low-concentration biomarker detection. The graphene/rGO dual-layer SPE demonstrated improved sensitivity compared with single-layer configurations, achieving a detection range from 0.2 pM to 55 nM and a detection limit of 2.398 pM. Error bars were calculated based on triplicates of each experiment, and the calibration plot of normalized current (I C/I blank) versus concentration (pM) is reliable with a linear regression coefficient (R^2^ = 0.97). This innovative design highlights the potential of graphene-based materials for rapid, label-free electrochemical immunosensors.

**Figure 5:**
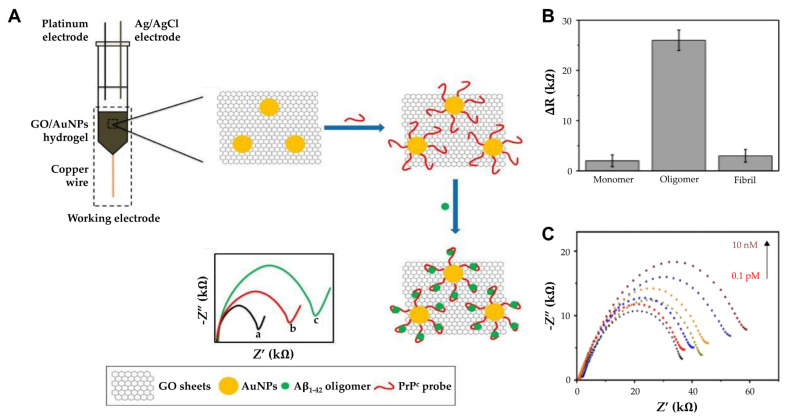
(**A**) The principle of AβO detection by GO/AuNPs -PrPC biosensor: The charge transfer resistance (Rct) of (a) bare GO/AuNPs electrode, (b) PrPC probe immobilized electrode, and (c) AβO captured GO/AuNPs -PrPC biosensor. (**B**) Comparison of ΔR for Aβ1–42 monomers, oligomers, and fibrils. (**C**) The impedance spectra of the GO/AuNPs -PrPC biosensor (black curve) and its response to CSF AβO with varying concentration (0.1 pM to 10 nM). (**A**–**C**): reproduced with permission from [79], published by International Journal of Nanomedicine 2018.

Further, incorporating hydrogels into biosensor designs has opened new avenues for enhanced sensitivity and specificity. Sun et al. used a hydrogel composed of graphene oxide (GO) and AuNPs. Unlike solid electrodes that can only adsorb biomolecules on the outer surface, they designed a soft graphene hydrogel electrode to allow biomolecules to rapidly penetrate and bind to the capture probe, thereby providing a 3D porous structure that facilitates the penetration and binding of biomolecules. (Figure 5A) [79]. The 3D porous hydrogel composed of GO and AuNPs provides a high surface area and enhanced electron transfer capabilities, while a PrPC peptide probe immobilized on AuNPs selectively binds Aβ oligomers for targeted detection. This structure enhances the electrochemical performance by amplifying electron transfer. Functionalized with a thiolated PrPC peptide probe, the biosensor exhibited high specificity toward AβOs in CSF or blood (Figure 5B). Moreover, the GO/AuNPs hydrogel contains approximately 96.19% water, which not only enhances biocompatibility but also provides a biomimetic environment that supports efficient target biomolecule binding. This combination of water content and functionalization with PrPC ensures a favorable environment for optimal sensor performance. The hydrogel’s biocompatibility and biomimetic environment further supported effective detection, achieving a detection limit of 0.1 pM and a linear range from 0.1 pM to 10 nM (Figure 5C). The GO/GNPs hydrogel stored at 4 °C was investigated by measuring the R ct daily, and the resistance did not show significant change for 8 days, suggesting that the electrode performance is not degraded and that repeated detection is possible. Such innovations underscore the utility of hydrogels in creating biosensors suitable for early-stage diagnosis of neurodegenerative diseases.

Alternatively, the use of antibody fragments has emerged as a promising approach to address the limitations of random antibody orientations, which can reduce antigen binding efficiency. Palla et al. developed an impedance-based immunosensor using antibody fragments to detect Aβ1-42 fibrils [80]. This improved the functionalization efficiency by compensating for the decrease in antigen binding efficiency due to the random adsorption of antibodies. The antibody fragments were then covalently bonded to the surface of the AuNPs-modified gold electrode via self-assembled 4,4′-thiobisbenzenethiol (TBBT). EIS was employed to monitor changes in Rct, with higher Rct values corresponding to increased antigen concentrations. This biosensor achieved a detection limit of 0.6 pM and a linear range from 0.5 pM to 4.0 pM in phosphate-buffered saline (PBS). This approach demonstrates the potential of antibody fragments to improve sensitivity and specificity in biomarker detection.

Parallel to immunosensors, aptamer-based biosensors have shown great promise for achieving high specificity in Aβ detection as well. Aptamer-based biosensors offer unparalleled specificity in biomarker detection due to their unique binding properties. Negahdary et al. developed an aptasensor using a fern leaves-like gold nanostructure (Au-FLGN) synthesized through electrodeposition [81]. The fern leaves-like gold nanostructure was synthesized through an electrodeposition process using polyethylene glycol 6000 (PEG 6000) as a shape-directing agent. The resulting nanostructure exhibited rough, spindle-like formations with sharp edges resembling fern leaves. This unique morphology provided an extensive surface area and high roughness factor, which were critical for enhancing aptamer immobilization and facilitating efficient electron transfer. The aptamer, a 107-mer thiol-modified RNA sequence, was designed for high specificity towards Aβ(1–42). The aptamer was then covalently immobilized onto the Au-FLGN surface through strong thiol–gold interactions. To prevent non-specific binding, mercapto-hexanol was applied to block the remaining uncoated areas of the gold surface. This immobilization strategy ensured a high surface density and optimal orientation of the aptamer, enabling efficient recognition and binding of Aβ. Detection was achieved using DPV, with ferro/ferricyanide serving as the redox system. The binding of the negatively charged Aβ peptide to the aptamer induced changes in the surface charge, reducing the accessibility of the redox marker. These changes were measured as decreases in the peak current, enabling highly sensitive detection of Aβ. The aptasensor demonstrated a linear detection range of 0.002 to 1.28 ng/mL and a remarkably low detection limit of 0.4 pg/mL. Three different Aβ concentrations were measured independently three times, resulting in a relative standard deviation (RSD) of 3.21%, demonstrating reproducibility and repeatability. Similarly, Zhang et al. developed an aptasensor incorporating a thiolated single-stranded DNA (ssDNA) aptamer immobilized on a gold rod electrode (AuR) [82]. Using EIS, this aptasensor achieved a detection limit of 0.03 nM and a linear range of 0.1–500 nM. These systems illustrate how aptamers can enhance biosensor performance, particularly in distinguishing neurotoxic oligomers from other amyloid species. The recent research on electrochemical biosensors for Amyloid-β is compared in Table 2.

## 4. Electrochemical Biosensor for Tau Proteins

Tau protein is an essential microtubule-associated protein that plays a critical role in stabilizing and maintaining the dynamics of microtubules in mature neurons [85,86]. It ensures proper cytoskeleton organization and facilitates axonal transport [87]. However, in pathological conditions such as PD and AD, Tau undergoes abnormal changes, including hyperphosphorylation and aggregation into NFTs [88,89]. These changes render the protein insoluble, leading to misfolding, destabilization of microtubules, impaired protein transport, and ultimately neuronal apoptosis [90,91]. Along with the toxic effects of aggregated Tau species, the loss of Tau’s normal function exacerbates neurodegenerative processes [92,93]. Given the close association of Tau pathology with the progression of neurological disorders, the development of highly sensitive, selective, and efficient diagnostic tools for detecting Tau biomarkers is of utmost importance. Recent advancements in nanotechnology have enabled the development of highly sensitive and selective electrochemical biosensors for detecting Tau protein, utilizing innovative nanomaterials to enhance detection capabilities.

**Figure 6:**
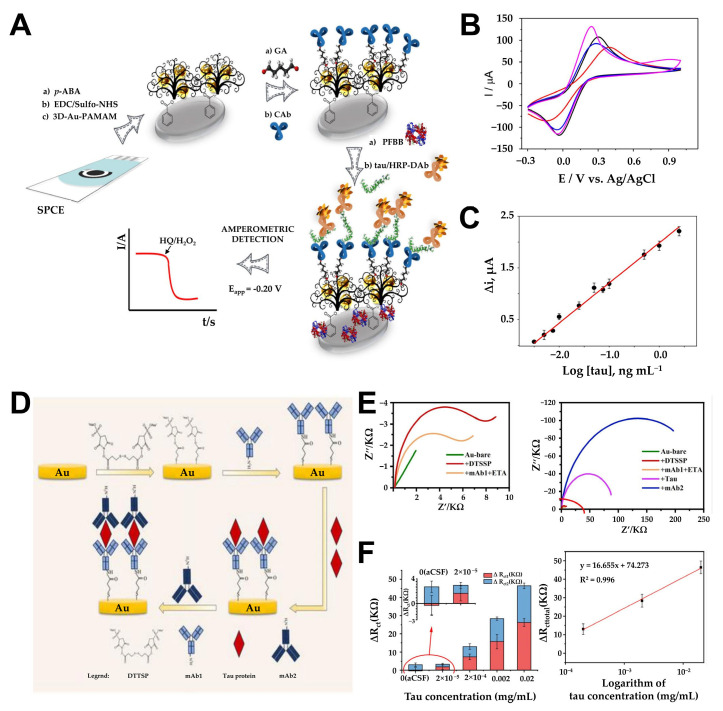
(**A**) Schematic illustration of the HRP-DAb-tau-CAb-3D-Au-PAMAM- p -ABA-SPCE immunosensor for Tau protein determination. (**B**) CVs recorded in 0.1 mol L^−1^ KCl aqueous solution containing 5 mM [Fe(CN)6]^3−/4−^ at a: bare SPCE (black line), HOOC-p-ABA-SPCE (red line), EDC/Sulfo-NHS-activated HOOC-p-ABA-SPCE (blue line), 3D-Au-PAMAM-p-ABA-SPCE (magenta line). CV: ν = 50 mV s^−1^. (**C**) Calibration plot constructed for the amperometric detection of Tau standard solutions with the HRP-DAb-tau-CAb-3D-Au-PAMAM-p-ABA-SPCE immunosensor. (**D**) Schematic diagram of sensor fabrication and detection process. (**E**) Nyquist plot of electrode surface modification with anti-Tau protein monoclonal antibody-1 (blue color symbol), Tau protein (red color symbol) binding, and anti-Tau protein monoclonal antibody-2 binding (navy color symbol). (**F**) Impedance value shift of different concentrations of Tau protein and linear relationship between impedance changes and Tau concentration. (**A**–**C**): reproduced with permission from [95], published by Biosensors and Bioelectronics 2020. (**D**–**F**): reproduced with permission from [96], published by Biosensors 2024.

Since aggregation and tangle formation of Tau protein play an important role in AD formation, Karaboga et al. developed a novel anti-Tau-based detection probe by depositing an rGO-AuNP nanocomposite onto the surface of a disposable ITO electrode [94]. In their approach, the rGO-AuNP platform facilitated antibody immobilization and the selective detection of Tau-441 protein, with its detection capabilities evaluated in CSF and serum. rGO was developed on ITO using a drop-casting method, and AuNPs were electrochemically deposited onto the functionalized ITO surface using CV in a potential range of −0.2 V to −1.3 V. The resulting rGO-AuNP nanocomposite acted as a signal-transducing element, mediating current flow or signaling analyte detection when integrated into the recognition element’s label. To enhance sensitivity, 11-mercaptoundecanoic acid (11-MUA) was employed as a covalent anchor, forming a self-assembled monolayer by incubating the ITO electrode in a solution of 11-MUA. Covalent bonds between the gold surface and -SH groups facilitated the immobilization process, while the -COO terminal was activated with EDC and NHS to immobilize anti-Tau antibodies, completing the electrode modification. EIS confirmed successful surface modifications, with the rGO-AuNP composite significantly reducing Rct due to its excellent conductivity. Upon measuring Tau-441 solutions of varying concentrations using EIS and CV, this biosensor demonstrated a linear detection range of 1–500 pg/mL and an LOD of 0.091 pg/mL, proving its suitability for analyzing Tau protein in CSF and serum. In addition, using six biosensor systems prepared at different times with the same procedure, the monitoring results showed similar linearity for Tau-441 and 1–500 pg/mL, and the relative standard deviation of the slope and intercept of the reproducibility were 3.02% and 3.41%, respectively. This led to the conclusion that repeatable measurements were possible. Similarly, Razzino et al. introduced a novel amperometric biosensor for Tau protein detection by functionalizing a screen-printed carbon electrode (SPCE) with p-aminobenzoic acid (p-ABA) via electropolymerization and incorporating a 3D-Au-PAMAM (polyamidoamine) nanocomposite (Figure 6A) [95]. This sensor utilizes a novel amperometric detection method using sandwich immunoassay and electrical transplantation. PAMAM dendrimers feature a three-dimensional structure, nanoscale size, low dispersity, high surface functionality, and flexibility, making them ideal nanomaterials for electrochemically compatible biosensors. The 3D-Au-PAMAM was covalently attached to the p-ABA-functionalized SPCE using EDC/Sulfo-NHS chemistry, with the nanocomposite serving as a scaffold for sandwich immunosensor development via crosslinking with glutaraldehyde (GA). Functionalizing SPCE with p-ABA introduced negatively charged carboxyl groups, reducing current flow; however, the addition of 3D-Au-PAMAM enhanced electron transfer rates due to Au’s superior conductivity properties (Figure 6B). Under optimized conditions, hydroquinone (HQ) was used as the electron transfer mediator, and H_2_O_2_ was added as the enzyme-substrate for amperometric detection of immune complex formation at −200 μA (Figure 6C). A linear range of 6–5000 pg/mL was achieved for Tau detection, with a calculated detection limit of 1.7 pg/mL. Building upon these advancements, Yang et al. presented an immune-impedance sensor for rapid Tau protein detection, utilizing a sandwich-based approach [96]. The system immobilized a primary monoclonal antibody (mAb1) on a self-assembled monolayer of 3,3′-dithiobis(sulfosuccinimidyl propionate) (DTSSP) on a gold electrode surface. The target antigen, Tau protein, is captured through antigen-antibody interactions, and signal amplification was achieved via a secondary monoclonal antibody (mAb2) (Figure 6D). Prior to Tau protein capture, the working electrode was immersed in a 1 mg/mL DTSSP solution, forming a SAM through thiol–gold chemical bonds. The mAb1 was subsequently added to the electrode surface and allowed to bind via alkyl sulfonate amino groups. Upon introducing Tau-441 protein and mAb2, a sandwich complex was formed. The specific binding of the secondary antibody to Tau protein formed a more compact self-assembled monolayer on the electrode surface, hindering charge transfer to the electrode surface and resulting in an increase in impedance response (Figure 6E). The performance of the functionalized sensor was evaluated by quantifying the total impedance change value using EIS measurements before and after the addition of Tau protein and mAb2. ΔRct1 represents the impedance difference before and after the addition of Tau protein, while ΔRct2 represents the impedance difference before and after the addition of mAb2. ΔRct_total indicates the total impedance change. This method demonstrated a linear detection range of 1 × 10^−4^ mg/mL to 0.01 mg/mL and an LOD of 1 × 10^−4^ mg/mL, validating its potential for detecting Tau in artificial cerebrospinal fluid (Figure 6F). Additionally, we verified the reproducibility of the experiment by obtaining a correlation coefficient of 0.998 in low-concentration Tau protein samples through triplicate repetition experiments.

In another innovative approach, Yola et al. designed an innovative electrochemical immunosensor platform using manganese sulfide nanoparticles/graphene oxide/polyaniline (MnS/GO/PANI) combined with magnetite-loaded gold nanoparticles (AuNP@Fe_3_O_4_) for the rapid detection of Tau protein [97]. This sensor utilized a MnS/GO nanocomposite formed via interactions between -COOH groups on GO and surface defects on MnS nanoparticles. MnS nanoparticles were covalently bound to GO via -NH group interactions and further stabilized by PANI. AuNP@Fe_3_O_4_ served as a signal amplifier; the synergistic effect of AuNPs and Fe_3_O_4_ resulted in the highest electrochemical signal. Using a sandwich-type immunosensor configuration with H_2_O_2_ as the redox probe, the system achieved a linear detection range of 0.1 pM–1 μM and an LOD of 0.01 pM. To demonstrate the selectivity, we performed repeated experiments (n = 6) to confirm that other proteins did not affect the highly selective electrochemical performance for Tau detection, and the current signal collected at the end of week 7 was about 99.08% of that at the end of week 1. These results suggest the excellent durability of the constructed electrochemical Tau immunosensor. Expanding on the concept of targeted detection for specific isoforms of Tau protein, Shui et al. introduced a sophisticated biosensor tailored for the Tau-381 isoform. This biosensor employs a dual-recognition system by combining aptamers and antibodies in a sandwich assay format [98]. AuNPs stabilized with cysteamine were utilized for signal enhancement due to their superior electron transfer properties. The biosensor construction begins with functionalizing a gold electrode using 3-mercaptopropionic acid (MPA) to form a self-assembled monolayer through covalent interactions between gold and sulfur. The stepwise modification of the gold electrode was validated through impedance spectroscopy, showing incremental increases in capacitance and resistance with each layer. This SAM introduces carboxylic acid groups on the electrode surface, allowing covalent immobilization of Tau-specific antibodies. The detection mechanism relies on the aptamer–antibody sandwich assay, where Tau-381-specific aptamers conjugated to AuNPs bind to the biomarker, forming a stable complex. DPV is used to monitor the resulting changes in electron transfer properties. The dual-recognition system ensures high specificity, while the signal amplification by AuNPs enables detection within a wide range from picomolar to femtomolar concentrations. Reproducibility was verified through replicate experiments (n > 3) using three human serum samples, obtaining RSD values of 4.8%, 5.2%, and 5.8% at a concentration of 0.43 pM, respectively.

**Figure 7:**
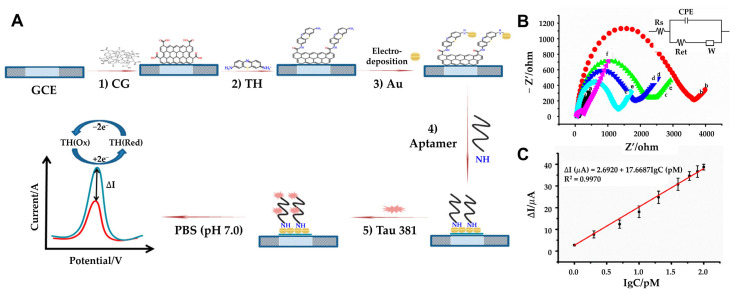
(**A**) Schematic representation of GCE/CG/TH/AuNPs/aptamer/Tau-381 aptasensor: (1) coating the GCE surface with CG, (2) followed by incubation with TH, (3) electrodeposition of HAuCl_4_·3H_2_O to AuNPs, (4) and Tau-381 (5) DPV evaluation of Tau-381 levels in human serum. (**B**) Impedance spectra of the electrode during modification: (a) CG/GCE (b) TH-CG/GCE (c) Au-TH-CG/GCE (d) Apt-Au-TH-CG/GCE (e) Tau-381-Apt-Au-TH-CG/GCE (f) in 10 mM [Fe(CN)6]^3−/4–^ (1:1 ratio). In the inset, Rs represents the solution resistance, W denotes the Warburg diffusion resistance, Ret corresponds to the electron-transfer resistance, and CPE signifies the double-layer capacitance. (**C**) Plot of ΔI vs. Log of Tau-381 concentration (n > 3). (**A**–**C**): reproduced with permission from [99], published by Biosensors 2019.

In contrast, Tao et al. developed a label-free electrochemical aptasensor incorporating carboxyl graphene (CG), thionin (TH), and AuNPs (Figure 7A) [99]. The use of CG provides high conductivity, a large surface area, and functional carboxyl groups, while TH acts as a redox signal generator and a bridge for AuNP deposition (Figure 7B). This sensor detects Tau-381 by monitoring steric hindrance and altered electron transfer caused by Tau binding, using DPV for signal analysis. With a detection range of 1.0 to 100 pM and an LOD of 0.7 pM, this platform offers an innovative and sensitive solution for Alzheimer’s diagnostics (Figure 7C). The reproducibility of the aptasensor was tested in triplicate. Five freshly prepared electrodes were used to detect the same concentration (10 pM) of Tau-381. The relative standard deviation (RSD) of the measurements was 4.9%, indicating that the aptasensor provides excellent sensor-to-sensor repeatability for Tau-381 detection. The recent research on electrochemical biosensors for Tau proteins is compared in Table 3.

## 5. Conclusions and Future Perspectives

Neurodegenerative diseases, such as Parkinson’s disease (PD) and Alzheimer’s disease (AD), represent a significant global health challenge due to their complex pathologies, overlapping biomarkers, and increasing prevalence. Despite advances in understanding these conditions, early and accurate diagnosis remains a critical bottleneck in improving patient outcomes. Key biomarkers like α-synucleins, amyloid-β, and Tau proteins have been widely studied, but conventional detection methods, such as enzyme-linked immunosorbent assays (ELISA) and polymerase chain reaction (PCR), are often invasive, time-intensive, and costly, limiting their clinical applicability.

In this regard, electrochemical biosensors have emerged as a transformative technology in neurodegenerative biomarker detection. One of the primary advantages of electrochemical biosensors is their high sensitivity and specificity, which allows for the detection of biomolecules at extremely low concentrations. This is particularly crucial in neurodegenerative diseases where early-stage biomarkers are present in extremely low concentrations. The ability to detect these biomarkers at the earliest stages could enable early diagnosis and intervention, potentially slowing disease progression and improving patient outcomes. Another significant advantage is the rapid response time. Unlike traditional detection methods, which require extensive sample preparation and prolonged processing times, electrochemical biosensors can provide near-real-time results. This capability is essential for timely decision-making in clinical settings and for effectively monitoring disease progression. Furthermore, these biosensors can be designed as portable and wearable devices via miniaturization, making them highly suitable for point-of-care applications.

The ease of fabrication of electrochemical biosensors and cost-effectiveness are also advantages. Traditional laboratory-based techniques for biomarker detection often require expensive reagents, specialized equipment, and trained personnel, limiting their accessibility. In contrast, electrochemical biosensors utilize low-cost electrode materials and nanomaterial-enhanced sensing platforms, reducing manufacturing costs while maintaining high performance. Additionally, electrochemical biosensors could exhibit excellent versatility, as they can be modified with various biorecognition elements, including antibodies, aptamers, enzymes, and molecularly imprinted polymers (MIPs). This flexibility allows for the design of highly selective biosensors tailored to detect multiple neurodegenerative disease biomarkers in complex biological fluids such as blood, cerebrospinal fluid (CSF), and saliva. Moreover, integrating these biosensors with nanomaterials enhances performance by improving signal amplification, biocompatibility, and surface functionalization, leading to enhanced detection capabilities. Given these advantages, electrochemical biosensors are considered a key technique to revolutionize neurodegenerative disease diagnostics, offering a sensitive, rapid, cost-effective, and portable alternative to conventional methods. However, despite their promise, several challenges and limitations must still be addressed to fully realize their clinical potential.

Looking to the future, the focus should shift toward the development of robust, multiplexed platforms capable of simultaneously detecting multiple biomarkers. This comprehensive approach can help differentiate between overlapping pathologies in neurodegenerative diseases, thereby supporting more accurate differential diagnoses and personalized treatment strategies. Furthermore, improving the long-term stability and biocompatibility of biosensors will facilitate their integration into routine healthcare, ensuring consistent performance over time. Additionally, efforts to simplify sensor fabrication and reduce production costs will also be vital to ensure that these advanced technologies are accessible and scalable for widespread clinical use. Another promising direction is the integration of electrochemical biosensors with advanced technologies, such as machine learning algorithms, artificial intelligence (AI), and wearable devices, which could open new avenues for real-time, point-of-care diagnostics. Such systems could enable continuous health monitoring, which is particularly beneficial for at-risk populations, and improve the dynamic tracking of disease progression and the efficacy of therapeutic interventions.

However, significant technical and regulatory challenges yet remain before electrochemical biosensors can be adopted in clinical settings. Reproducibility and standardization are major hurdles, as even minor variations in sensor fabrication, electrode modifications, and biomolecule immobilization can lead to inconsistent results. Addressing this issue requires the development of standardized fabrication techniques and universal calibration protocols to ensure reliable data across different laboratories and healthcare facilities. The complexity of biological samples also presents a challenge, as they contain numerous interfering substances that may cause non-specific binding and background noise in electrochemical measurements. Future biosensors must incorporate highly selective nanostructured interfaces and dual-mode sensing strategies to enhance specificity and minimize false signals. Additionally, ensuring regulatory approval and clinical validation is critical. Unlike traditional diagnostic methods, electrochemical biosensors must undergo extensive clinical testing to demonstrate their accuracy, sensitivity, and robustness.

Despite these challenges, the future of electrochemical biosensors in neurodegenerative disease detection is still promising. With continued interdisciplinary collaboration and technological innovation, electrochemical biosensors have the potential to transform early detection, disease monitoring, and therapeutic management of neurodegenerative disorders, paving the path for more effective, accessible, and personalized healthcare solutions.

## Figures and Tables

**Table 1:** Comparison of linear ranges and detection limits according to a-synuclein detection used nanomaterials.

Sensor Platform	Sensor Type	Detection Technique	Linear Range	LOD	Reference
AuNPs, ZnO NSs	Immunosensor	CV, EIS	0.5–10 pg mL^−1^	0.08 pg mL^−1^	[63]
AuNPs, polyglutaminic acid		SWV	4–2000 pg mL^−1^	0.135 pg mL	[52]
AuNPs, PDG, MWCNTs, rGO		SWV	0.05 fM–500 fM	0.03 fM	[64]
AuNPs, MNP		EIS	1–1000 ng/mL^−1^	310 pg mL^−1^	[67]
AuNPs		DPV (Differential pulse voltammetry), SWV, ChA (chronoamperometry)	4–64 ng mL^−1^	4 ng mL^−1^	[53]
AuNPs, graphene		ChA, EIS	4–128 ng mL^−1^	4 ng mL^−1^	[14]
AuNSs, pTH, ssDNA	aptasensor	EIS	0.10 aM–10.00 fM	0.07 aM	[65]
AuNPs, GDY, DA/MBA/WSe2		CV, EIS	10 aM–1.0 nM	3.3 aM	[66]
carbon nanofibers, zeolitic imidazolate framework nanosheets		EIS	1 fg mL^−1^–0.2 ng mL^−1^ (52.6 fM–0.1 nM)	0.87 fg mL^−1^ (45.7 fM)	[68]
Peptide-imprinted polymers	Molecularly imprinted polymer	CV	0.065 pM to 0.65 nM	4.0 pM	[69]

**Table 2:** Comparison of linear ranges and detection limits according to amyloid-beta detection used nanomaterials.

Sensor Platform	Sensor Type	Detection Technique	Linear Range	LOD	Reference
uNP, TBBT	immunosensor	EIS	0.5 pM–4.0 pM	0.64 pM	[80]
dual layer graphene, rGO		DPV	11 pM–55 nM	2.398 pM	[78]
AuNP, chitosan, CNT		DPV	10.0 pg mL–100.0 ug mL	0.87 pg mL	[77]
AuNP, RNA	aptasensor	DPV	0.002–1.28 ng mL^−1^	0.4 pg mL^−1^ (88.6 amol L^−1^)	[81]
AuNP, GO, Thiolated PrP		EIS	0.1 pM–10 nM	0.1 pM	[79]
AuD, ssDNA		EIS	0.1–500 nM	0.03 nM	[82]
poly(pyrrole-3-carboxylic acid), PrP		EIS	10^−9^–10 nM	1 aM	[76]
AgNP, SiO2	MIP	DPV	5 pg mL^−1^–10 ng mL^−1^	1.22 pg mL^−1^	[83]
titanium carbide Mxene, multi-walled carbon nanotubes		DPV	1.0 fg mL^−1^–100.0 fg mL^−1^	0.3 fg mL^−1^	[84]

**Table 3:** Comparison of linear ranges and detection limits according to Tau protein detection used nanomaterials.

Sensor Platform	Sensor Type	Detection Technique	Linear Range	LOD	Reference
MnS/GO/PANI, AuNP@Fe3O4	immunosensor	DPV	0.1 pM–1.0 μM	0.01 pM	[97]
AuNP, PAMAM dendrimers		CV, EIS, CA	6–5000 pg mL (0.11–91 pM)	0.031 pM (1.7 pg mL)	[95]
rGO, AuNPs, 11-MUA		CV, EIS, SFI (Single Frequency Impedance)	1–500 pg/mL	0.091 pg/mL	[94]
DTSSP		CV, EIS	1 × 10^−4^ mg mL^−1^ to 0.01 mg mL^−1^	1 × 10^−4^ mg mL^−1^	[96]
lipoic acid, n-butylamine, hexanethiol		EIS	-	-	[100]
AuNPs	aptasensor	DPV	0.5 pM–100 pM	0.42 pM	[98]
AuNPs, carboxyl graphene, thionin		DPV	1.0 pM–100 pM	0.70 pM	[99]
